# Advancing the Understanding of Restless Legs Syndrome in Latin America: A Response to Lopes et al.

**DOI:** 10.1055/s-0046-1825531

**Published:** 2026-09-02

**Authors:** Karem Parejo, Ulises Jimenez-Correa, Lourdes M. DelRosso

**Affiliations:** 1 Shaio Clinic Foundation, Bogota, Colombia.; 2 Facultad de Medicina, National Autonomous University of Mexico, Mexico City, Mexico.; 3 Department of Medicine, Division of Pulmonary and Sleep Medicine, University of California, San Francisco, Fresno, CA, United States.

We thank Lopes et al. for their thoughtful comments 1 regarding our article discussing the American Academy of Sleep Medicine's (AASM) guidelines for the treatment of restless legs syndrome (RLS) in the context of Latin America. 2 Their letter raises several important considerations related to epidemiology, diagnostic interpretation, and language in the characterization of RLS. We welcome this opportunity to further clarify the intent and scope of our work.

A key point to emphasize is the scope of the original article. Our publication was not intended to serve as a comprehensive review of the epidemiology or diagnostic complexities of RLS in Latin America. Rather, the purpose of the article was to highlight how the recently updated AASM treatment guidelines for RLS may intersect with the practical realities of clinical care across Latin American settings. 3 These realities include differences in medication availability (and cost-related accessibility), variability in healthcare infrastructure (and insurance issues), and the influence of cultural and regional practices on therapeutic approaches. 4 For example, a publication that studied gabapentinoid consumption in 65 countries and regions from 2008 to 2018 found an increase in sales of pregabalin and gabapentin in the data from all included countries, except for Venezuela—which was attributed to its economic downturn and reduced health expenditure. 5 Our goal was, therefore, to raise awareness of the diversity of clinical practice in the region and to stimulate discussion about how evidence-based international guidelines may be interpreted and implemented in different healthcare environments.

Lopes et al. appropriately note that prevalence estimates of RLS in Latin America vary widely and that methodological differences across studies may contribute to this variability. We agree that epidemiologic estimates depend heavily on diagnostic criteria, study design, and the instruments used to identify symptoms in population studies. 6 The reference to prevalence data in our article was intended to illustrate the range of findings reported in the literature, rather than to establish definitive estimates of RLS prevalence in any specific country. As highlighted by the authors, the interpretation of questionnaire-based diagnostic approaches and the potential overlap with other sleep or neurological conditions remain important considerations in epidemiologic research.

Their comments regarding diagnostic language and the interpretation of criteria also highlight an important broader issue: the complexity of translating clinical definitions across different cultural and linguistic contexts. In the case of RLS in Mexico, the main reason of consultation is “desesperación”, despair, in the legs that prevents one from falling asleep; therefore, the translation of the word “restless” into Spanish does not correspond to the reason for the patients' consultation. This challenge is particularly relevant in regions where diagnostic tools and clinical terminology may be adapted for different languages and healthcare systems. Continued dialogue and methodological refinement in epidemiologic research will be essential to ensure accurate characterization of RLS across diverse populations.

At the same time, we would like to reiterate that the central focus of our article was treatment and clinical management rather than epidemiology. The AASM guidelines provide an important evidence-based framework for the management of RLS, including recommendations regarding pharmacologic therapies, iron replacement strategies, and considerations related to augmentation. However, the implementation of these recommendations may be influenced by regional factors, such as medication availability, regulatory approval, healthcare access, and local prescribing practices. In some parts of Latin America, clinicians may face limitations in access to certain medications, or incorporate alternative therapeutic strategies that reflect local clinical experience and resource availability.

In this sense, the discussion raised by Lopes et al. 1 underscores an important point that aligns with the broader intention of our article: the need for ongoing regional dialogue regarding how international guidelines are applied in diverse healthcare contexts. While the AASM guidelines represent an important global reference for the treatment of RLS, complementary regional perspectives may help inform how these recommendations are adapted to the demographic characteristics and population health status, healthcare systems, and cultural practices present in Latin America.

Ultimately, our intention was to bring attention to these practical considerations and to encourage greater discussion regarding the implementation of evidence-based RLS management in the region. We appreciate the opportunity to engage in this dialogue and agree that continued collaborative research will be essential to advance understanding of RLS and to improve care for affected patients across Latin America.
